# Prevalence of Subthreshold Hypomania and Impact on Internal Validity of RCTs for Major Depressive Disorder: Results from a National Epidemiological Sample

**DOI:** 10.1371/journal.pone.0055448

**Published:** 2013-02-06

**Authors:** Nicolas Hoertel, Yann Le Strat, Frédéric Limosin, Caroline Dubertret, Philip Gorwood

**Affiliations:** 1 Assistance Publique-Hôpitaux de Paris (AP-HP), Hôpital Corentin-Celton, Service de Psychiatrie, Issy-les-Moulineaux, France; 2 Université Paris Descartes, PRES Sorbonne Paris Cité, Paris, France; 3 INSERM U894, Centre Psychiatrie et Neurosciences, Paris, France; 4 Assistance Publique-Hôpitaux de Paris (AP-HP), Hôpital Louis Mourier, Service de Psychiatrie et Addictologie, Colombes, France; 5 Groupe Hospitalier Sainte-Anne, CMME, Paris, France; University of Iowa Hospitals & Clinics, United States of America

## Abstract

**Background:**

Growing evidence supports the validity of distinguishing major depressive disorder (MDD) plus a lifetime history of subthreshold hypomania (D(m)) from pure MDD in psychiatric classifications. The present study sought to estimate the proportion of individuals with D(m) that would have been included in RCTs for MDD using typical eligibility criteria, and examine the potential impact of including these participants on internal validity.

**Methods:**

Data were derived from the 2001–2002 National Epidemiological Survey on Alcohol and Related Conditions (NESARC), a national representative sample of 43,093 adults of the United States population. We examined the proportion of participants with a current diagnosis of pure MDD and D(m) that would have been eligible in clinical trials for MDD with a traditional set of eligibility criteria, and compared it with that of participants with bipolar 2 disorder if the same set of eligibility criteria was applied. We considered 4 models including different definitions of subthreshold hypomania.

**Results:**

We found that more than 7 out of ten participants with pure MDD and with D(m) would have been excluded by at least one classical eligibility criterion. Prevalence rate of individuals with D(m) in RCTs for MDD with traditional eligibility criteria would have ranged from 7.98% to 22.59%. Overall exclusion rate of individuals with MDD plus at least 4 lifetime concomitant hypomanic probes significantly differ from those with pure MDD, whereas it was not significantly different in those with at least 2 lifetime concomitant hypomanic probes compared to those with bipolar 2 disorder.

**Conclusions:**

The current design of clinical trials for MDD may suffer from impaired external validity and potential impaired internal validity, due to the inclusion of a substantial proportion of individuals with subthreshold hypomania presenting with similar pattern of exclusion rates to those with bipolar 2 disorder, possibly resulting in a selection bias.

## Introduction

The practice of evidence-based medicine is generally understood as the application to clinical care of knowledge derived from double blind, randomized placebo-controlled trials (RCTs) [1], [2]. However, emerging data indicate that restrictive eligibility criteria used by RCTs to reach high internal validity (i.e., highly homogeneous samples) is achieved at the cost of diminished external validity (i.e., applicability of clinical trial results to routine clinical care) [3], [4] [5], perpetuating the gap between research and clinical practice [6].

Major depressive disorder (MDD) is considered to be the most prevalent psychiatric disorder, with considerable functional and social impairment [7], [8]. Growing clinical and epidemiologic evidence indicates that at least part of the heterogeneity observed in MDD is due to the high prevalence of bipolar features, supporting the validity of distinguishing MDD plus a lifetime history of subthreshold hypomania (D(m)) from pure MDD (i.e., MDD without a lifetime history of subthreshold hypomania) in psychiatric classifications, recently acknowledged in the posted DSM-5 update [9], [10]. Previous studies conducted in both clinical and general population [9]–[15] suggest that the prevalence of lifetime history of subthreshold hypomania in individuals with MDD ranges from 30% to 55%, supporting the existence of large overlaps between unipolar and bipolar disorders.

The recognition of subthreshold hypomania is important for different reasons, since depressed individuals with a lifetime history of subthreshold hypomania have greater rates of comorbidity than those without such a condition [9]–[11], [13], [15], [16], including higher rate of family history of mania and younger age at onset [9], [11], [15], [16], increased risk for suicide [9]–[11], [17], greater rate of mixed episodes and mania/hypomania during antidepressant therapy [16], and higher conversion rate to threshold-level bipolar disorder [15], [18]. Therefore, subthreshold bipolarity may be the source of a selection bias and influence treatment outcomes in RCTs for MDD.

Eligibility criteria may preferentially impact subjects with D(m) in RCTs for MDD. Examining the prevalence of individuals with D(m) enrolled in clinical trials for MDD is required, and may help estimating the potential impact on internal validity as well as guiding eligibility criteria operationalization for future clinical trials in major depressive disorder.

Because most RCTs examine separately efficacy of treatments for major depressive disorder and bipolar depression, the present study assessed the effect of applying exclusion criteria commonly used in clinical trials for major depressive disorder to a large (n = 43,093), nationally representative of the U.S. general population sample, the National Epidemiological Survey on Alcohol and Related Conditions (NESARC). Our aims were 1) to estimate the proportion of individuals with D(m) that would have been included in RCTs for MDD using classical eligibility criteria, and 2) examine the potential impact of including these patients on internal validity of RCTs for MDD. We first determined the prevalence of D(m) (i.e., MDD plus a lifetime history of subthreshold hypomania) and pure MDD in the NESARC. We applied a standard set of exclusion criteria commonly used in clinical trials for MDD, using a method previously described by Blanco and colleagues in clinical trials for major depression [3]. We then examined the proportion of all participants with a current diagnosis of D(m) and pure MDD in the NESARC that would have been eligible if the traditional eligibility criteria were applied to these samples, and compared it with that of individuals with bipolar 2 disorder if they were applied the same set of eligibility criteria. Because no consensus subthreshold bipolar-specifier diagnosis is available to date [11], [19], we defined four models including different subthreshold bipolar-specifier diagnoses. We hypothesized that 1) a significant proportion of subjects that would have been eligible for clinical trials for MDD present with a lifetime history of subthreshold hypomania, and 2) a substantial proportion of individuals with D(m) significantly differ from those with pure MDD but not from those with bipolar 2 disorder in overall eligibility rate, assuming that they share a similar pattern of exclusion rates. Because individuals who seek treatment for a disorder may differ from those who do not [3], [20], [21], we applied the exclusion criteria first to all participants with a current diagnosis of D(m) and pure MDD, and then to the subsamples of participants who sought treatment.

We used the NESARC for our study because it is the largest representative survey with information on major depressive disorder in U.S. adults. By employing this large representative sample, we sought to stress the consequences of including participants with D(m) in clinical trials for MDD, resulting in a potential selection bias, within a broad public health context.

## Methods

### NESARC Sample

The 2001–2002 NESARC is a nationally representative survey of the population of the United States conducted by the U.S. Census Bureau under the direction of the National Institute on Alcoholism and Alcohol Abuse (NIAAA), and described in detail elsewhere [22]. The NESARC target population was the civilian noninstitutionalized population, aged 18 years and older, residing in households and group quarters in the 50 states and the District of Columbia. Data collection was conducted via face-to-face computer assisted personal interviews under the supervision of the NIAAA staff. The resulting sample size was 43,093 and the overall survey response rate was 81%. African Americans, Hispanics, and young adults (aged 18–24) were oversampled. Once weighted, the data were adjusted to be representative of the U.S. population for various sociodemographic variables, based on the 2000 Decennial. Rights to confidentiality of NESARC participants were carefully protected. All NESARC participants provided written informed consent and were assured that their participation was voluntary. The research protocol, including informed consent procedures, received full ethical review and approval from the U.S. Census Bureau and the Office of Management and Budget [22].

### DSM-IV Diagnostic Interview

Lifetime and twelve-month psychiatric diagnoses were made according to the DSM-IV criteria with the Alcohol Use Disorder and Associated Disabilities Interview Schedule-DSM-IV Version (AUDADIS-IV), a valid and reliable fully structured diagnostic interview designed for use by professional interviewers who are not clinicians [22], [23]. The test-retest reliability [24], [25] of the AUDADIS-IV diagnosis of MDE are good (κ = 0.64–0.67), and a clinical reappraisal study [26] of major depression indicated good agreement between AUDADIS-IV and psychiatrist diagnoses (κ = 0.64–0.68). The Reliability of the AUDADIS-IV in assessing DSM-IV anxiety (κ = 0.40–0.60) and personality disorders (κ = 0.40–0.67) was fair to good [25], [26], and good to excellent for substance use disorders (κ = 0.54–0.76) [22], [25], [27], [28].

### Mood Disorders Assessment

Lifetime and twelve-month mood disorders were diagnosed following the DSM-IV criteria, except for the requirement of symptoms assessing a mixed episode (criterion B for major depressive disorder and criterion C for hypomania). Consistent with the DSM-IV diagnosis guidelines, a major depressive episode (MDE) was diagnosed when an individual reported at least 2 weeks of persistent depressed mood or anhedonia, accompanied by a total of at least 5 of the 9 DSM-IV symptoms of MDE during the episode. Major depressive disorder (MDD) was defined as having a lifetime history of at least 1 MDE, without a lifetime history of mania or hypomania. Participants reporting a major depressive episode occurring during the year preceding the interview without any lifetime history of mania or hypomania were considered as having a current major depressive disorder (MDD). Participants with current MDD who declared “going anywhere or saw anyone to get help for low mood” during the year preceding the interview were considered as seeking treatment.

Consistent with a prior research [10], criteria for subthreshold hypomania diagnosis included the lifetime presence of at least one of the three screening questions for the criterion A for hypomania: (i) “In your entire life, have you ever had a time lasting at least 1 week when you felt so extremely excited, elated or hyper that other people thought you weren’t your normal self ?” or (ii) “In your entire life, have you ever had a time lasting at least 1 week when you felt so extremely excited, elated or hyper that other people were concerned about you ?” or (iii) “In your entire life, have you ever had a time lasting at least 1 week when you were so irritable or easily annoyed that you would shout at people, throw or break things, or start fights or arguments ?”, and failure to meet the full diagnostic criteria for mania or hypomania. Participants who endorsed either of these questions were then asked an extensive list of symptom questions that operationalize DSM-4 criterion B for hypomania. Because no consensus subthreshold bipolar-specifier diagnosis is available to date [11], [19], we defined 4 models including different definitions of subthreshold hypomania. Among participants with current MDD, those who endorsed at least 1, 2, 3 or 4 lifetime concomitant hypomanic probes screening criterion A or B for hypomania were successively defined as having a current diagnosis of MDD plus a lifetime history of subthreshold hypomania (D(m)). By contrast, those without a lifetime history of subthreshold hypomania were successively classified as having a current diagnosis of pure MDD across the 4 models used.

In each of the four models, participants with a current diagnosis of MDD were divided in 2 mutually exclusive subgroups as follows: 1) current pure MDD (without a lifetime history of subthreshold hypomania, hypomania or mania), 2) current MDD plus a lifetime history of subthreshold hypomania (D(m)). In Model 1, pure MDD was defined as having no lifetime history of hypomanic probe, whereas D(m) was defined as having at least 1 lifetime hypomanic probe screening criterion A or B for hypomania. In Model 2, pure MDD was defined as having 0 or 1 lifetime history of hypomanic probe, whereas D(m) was defined as having at least 2 lifetime concomitant hypomanic probes screening criterion A or B for hypomania. In Model 3, pure MDD was defined as having 0, 1, or 2 lifetime history of hypomanic probes, whereas D(m) was defined as having at least 3 lifetime concomitant hypomanic probes. At last, in Model 4, pure MDD was defined as having 0, 1, 2, or 3 lifetime history of hypomanic probes screening criterion A or B for hypomania, whereas D(m) was defined as having at least 4 lifetime concomitant hypomanic probes (e.g., 3 criterion A probes plus at least one criterion B probe, or 2 criterion A probes plus at least 2 criterion B probes). Mood disorders were primary in the analyses (or “independent”, i.e., general medical condition or substance-induced mood disorders were ruled out).

### Clinical Trials Eligibility Criteria

Exclusion criteria commonly used in clinical trials for major depressive disorder were applied to a sample representative of the general population, the NESARC, to examine the proportion of individuals with a current DSM-IV diagnosis of MDD that would have been eligible for a typical clinical trial. We used traditional efficacy eligibility criteria proposed by Zimmerman and colleagues [29], because they constitute the best available representative set of exclusion criteria used in clinical trials for MDD. These criteria are presented in Table 1. In order to reproduce a clinical trial with typical exclusion criteria, we applied these traditional efficacy eligibility criteria to all individuals with a 12-month DSM-IV diagnosis of pure MDD, and to those with D(m) within the last 12 months, and then to the subsamples of participants who had sought treatment for depression, in the NESARC sample. The percentages of individuals excluded by criteria 1 through 4, and 6 through 7 were estimated from data collected by the AUDADIS-IV. Criterion 2 “significant risk of suicide” was considered met if the person reported suicide attempt within the last year, the timeframe used by the AUDADIS-IV when assessing the presence of “current” symptoms. Criterion “alcohol/drug use disorder” was approximate using a 12-month rather than 6-month time frame. Information to approximate criteria 5 and 8 was not available in the NESARC.

**Table 1:** Estimated Percentages of Adults with current D(m) and Pure MDD in NESARC excluded from Typical Clinical Trials of Treatments for DSM-IV MDD by Traditional Eligibility Criteria.

	MDD Subgroups								D(BP2)
	Pure MDD				D(m)				
	Model				Model				
	1 (n = 1,807)	2 (n = 1,865)	3 (n = 1,961)	4 (n = 2,066)	1 (n = 527)	2 (n = 469)	3 (n = 373)	4 (n = 268)	(n = 247)
Traditional efficacy eligibility criteria^a^	% (SE)	% (SE)	% (SE)	% (SE)	% (SE)	% (SE)	% (SE)	% (SE)	% (SE)
1- Current psychotic features	1.34 (0.34)	1.30 (0.33)	1.31 (0.32)	1.33 (0.31)	1.24 (0.36)	1.40 (0.40)	1.39 (0.43)	1.26 (0.48)	1.26 (0.71)
2- Significant risk of suicide	8.96 (0.91)	9.26 (0.93)	9.52 (0.89)	9.20 (0.85)	16.13 (1.93)	15.90 (1.98)	16.31 (2.23)	22.18 (3.18)	12.84 (2.61)
3- Alcohol/drug use disorder inthe last year	15.62 (0.91)	15.50 (0.90)	15.76 (0.89)	15.70 (0.89)	17.65 (2.07)	18.34 (2.31)	17.77 (2.72)	19.22 (3.29)	28.55 (3.59)
4- Comorbid dysthymic disorder	15.17 (1.09)	15.50 (1.09)	15.21 (1.05)	15.47 (1.05)	18.42 (1.96)	17.59 (2.17)	19.58 (2.57)	19.66 (2.70)	14.33 (2.44)
5- Score <18 on HAM-D	NA	NA	NA	NA	NA	NA	NA	NA	NA
6- Past-year comorbid anxiety disorders^b^	35.59 (1.36)	35.22 (1.36)	34.91 (1.33)	35.01 (1.28)	37.99 (2.75)	39.72 (2.93)	42.43 (3.36)	45.31 (3.82)	37.67 (3.84)
7- Episode duration of <4 weeksor >2 years	39.67 (1.54)	39.97 (1.50)	39.33 (1.44)	38.94 (1.40)	37.92 (2.69)	36.53 (2.90)	38.85 (3.37)	41.78 (3.77)	46.74 (3.52)
8- Borderline personality disorder	NA	NA	NA	NA	NA	NA	NA	NA	NA
Excluded by at least one criterion	71.96 (1.32)	71.94 (1.31)	71.57 (1.27)	71.32 (1.25)	73.41 (2.44)	73.67 (2.64)	75.99 (3.10)	80.16 (3.29)	81.69 (2.99)
Eligible participants	28.04 (1.32)	28.06 (1.31)	28.43 (1.27)	28.68 (1.25)	26.59 (2.44)	26.33 (2.64)	24.01 (3.10)	19.84 (3.29)	18.31 (2.99)

a Derived from Zimmerman et al. [29] (method described in the paper).

b Includes panic disorder, agoraphobia, social anxiety disorder, specific phobia, and generalized anxiety disorder.

Percentages are weighted values.

Abbreviations: MDD = Major Depressive Disorder, D(m) = MDD plus Subthreshold Hypomania, D(BP2) = Bipolar 2 depressive disorder, HAM-D = Hamilton Rating Scale for Depression, NESARC = National Epidemiological Survey on Alcohol and Related Conditions, NA = information not available in NESARC.

Models 1, 2, 3 and 4 indicates that subthreshold hypomania was defined as having respectively at least 1, 2, 3, or 4 lifetime concomitant hypomanic probes screening criterion A or B for hypomania.

### Statistical Analysis

We first determined the percentage (and 95% confidence interval) of survey participants with a current DSM-IV diagnosis of pure MDD and D(m) who would have been excluded by individually applying each exclusion criterion in clinical trials. Because individuals might have been excluded by more than one criterion, we also calculated the overall percentage of subjects who would have been excluded by the simultaneous application of all criteria. In each model, we conducted these analyses for all participants with a current DSM-IV diagnosis of pure MDD and D(m). The same criteria were applied to individuals with a current bipolar 2 depressive disorder to examine potential differences in the pattern of exclusion rates between these individuals and those with D(m). We conducted these analyses for all individuals with a current diagnosis of pure MDD, MDD plus a lifetime history of subthreshold hypomania, bipolar 2 depressive disorder, and for the subsamples of individuals who had sought treatment for depression, according to the four subthreshold bipolar-specifier diagnoses defined above.

Because of the weighting and clustering used in the NESARC design, all statistical analyses were performed using the Taylor series linearization method, a design-based method implemented using SUDAAN, version 10 (RTI International, Research Triangle Park, N.C.). Significance tests of sets of coefficients were performed using Wald chi-square tests based on design-corrected coefficient variance-covariance matrices. Statistical significance was evaluated using a two-sided design with alpha set at 0.05.

## Results

Out of the 3,119 individuals reporting a twelve-month MDE, 2,334 had a major depressive disorder and 785 (25.27%, SE = 0.98) a bipolar depression. In the subsample of individuals who had sought treatment (n = 1,359), 972 had a diagnosis of MDD and 387 (28.77%, SE = 1.58) a diagnosis of bipolar depression.

The 12-month prevalence rates of DSM-IV MDD, MDD plus a lifetime history of subthreshold hypomania, and pure MDD were respectively 5.87% (SE = 0.17, n = 2,602), 1.84% (SE = 0.09, n = 795) and 4.04% (SE = 0.12, n = 1,807), when using the less stringent bipolar-specifier diagnosis. Nearly one third (31.27%, SE = 1.18, n = 795) of the participants with a 12-month diagnosis of MDD reported at least one lifetime hypomanic probe.

The percentage of participants currently presenting with major depressive disorder that would have been excluded by at least 1 out of the 6 traditional and available criteria in clinical trials for MDD ranged respectively from 71.32% to 71.96% in participants with pure MDD, and from 73.41% to 80.16% in those with D(m), according to the model used (Table 1). This percentage rose respectively from 73.79% to 74.31% and from 79.35% to 81.54% in the seeking-treatment subsamples of participants with pure MDD and D(m) (Table 2).

**Table 2:** Estimated Percentages of Adults with current D(m) and Pure MDD in NESARC Seeking Treatment for depression excluded from Typical Clinical Trials of Treatments for DSM-IV MDD by Traditional Eligibility Criteria.

	MDD Subgroups								D(BP2)
	Pure MDD				D(m)				
	Model				Model				
	1 (n = 725)	2 (n = 748)	3 (n = 795)	4 (n = 844)	1 (n = 247)	2 (n = 224)	3 (n = 177)	4 (n = 128)	(n = 87)
Traditional efficacy eligibility criteria^a^	% (SE)	% (SE)	% (SE)	% (SE)	% (SE)	% (SE)	% (SE)	% (SE)	% (SE)
1- Current psychotic features	2.60 (0.78)	2.51 (0.76)	2.55 (0.75)	2.53 (0.71)	2.25 (0.74)	2.48 (0.82)	2.32 (0.84)	2.32 (0.95)	2.45 (1.63)
2- Significant risk of suicide	12.63 (1.56)	12.70 (1.53)	13.71 (1.50)	13.27 (1.45)	25.51 (3.34)	26.61 (3.56)	25.87 (3.82)	34.32 (5.01)	17.91 (5.12)
3- Alcohol/drug use disorder inthe last year	16.56 (1.53)	16.49 (1.51)	16.87 (1.48)	17.46 (1.52)	18.71 (3.15)	19.13 (3.46)	18.19 (4.06)	14.79 (3.59)	24.26 (5.53)
4- Comorbid dysthymic disorder	21.97 (2.05)	21.95 (1.99)	21.31 (1.90)	21.64 (1.96)	23.14 (3.26)	23.31 (3.55)	26.47 (4.31)	26.56 (4.55)	19.00 (4.56)
5- Score <18 on HAM-D	NA	NA	NA	NA	NA	NA	NA	NA	NA
6- Past-year comorbid anxiety disorders^b^	41.64 (2.24)	41.19 (2.20)	41.25 (2.13)	41.21 (2.07)	44.24 (4.13)	45.97 (4.44)	47.01 (4.76)	49.81 (5.23)	53.85 (6.39)
7- Episode duration of <4 weeksor >2 years	37.33 (2.11)	37.47 (2.08)	36.98 (2.00)	36.75 (1.94)	32.81 (3.88)	31.92 (3.96)	32.59 (4.55)	32.14 (4.90)	34.68 (5.56)
8- Borderline personality disorder	NA	NA	NA	NA	NA	NA	NA	NA	NA
Excluded by at least one criterion	73.79 (1.94)	73.80 (1.89)	73.96 (1.83)	74.31 (1.73)	79.35 (3.00)	79.90 (3.09)	80.82 (3.49)	81.54 (3.90)	85.37 (4.42)
Eligible participants	26.21 (1.94)	26.20 (1.89)	26.04 (1.83)	25.69 (1.73)	20.65 (3.00)	20.10 (3.09)	19.18 (3.49)	18.46 (3.90)	14.63 (4.42)

a Derived from Zimmerman et al. [29] (method described in the paper).

b Includes panic disorder, agoraphobia, social anxiety disorder, specific phobia, and generalized anxiety disorder.

Percentages are weighted values.

Abbreviations: MDD = Major Depressive Disorder, D(m) = MDD plus Subthreshold Hypomania, D(BP2) = Bipolar 2 depressive disorder, HAM-D = Hamilton Rating Scale for Depression, NESARC = National Epidemiological Survey on Alcohol and Related Conditions, NA = information not available in NESARC.

Models 1, 2, 3 and 4 indicates that subthreshold hypomania was defined as having respectively at least 1, 2, 3, or 4 lifetime concomitant hypomanic probes screening criterion A or B for hypomania.

The criterion leading to the highest exclusion rate was respectively having a any past-year comorbid anxiety disorder for the full sample and the treatment-seeking subsample of participants with D(m) as well as for the treatment-seeking subsample of participants with pure MDD, and having an episode duration lower than 4 weeks or higher than 2 years for the full sample of individuals with pure MDD (Tables 3 and 4). Participants with MDD plus at least 3 lifetime concomitant hypomanic probes were significantly more likely to report any past-year anxiety disorder than those with pure MDD or MDD plus 1 or 2 lifetime concomitant hypomanic probes in the full sample. Significant risk of suicide was significantly more prevalent in individuals with MDD plus at least 4 lifetime concomitant hypomanic probes compared with those with MDD without such a condition in the full sample and in the treatment-seeking subsample.

**Table 3:** Comparisons of Exclusion Rates between Adults with current D(m), Pure MDD, and D(BP2) in Typical Clinical Trials of Treatments for DSM-IV MDD by Traditional Eligibility Criteria.

	D(m) vs. Pure MDD								D(m) vs. D(BP2)						
	Model								Model						
	1		2		3		4		1		2		3		4
Traditional efficacy eligibilitycriteria^a^	OR [95%CI]		OR [95%CI]		OR [95%CI]		OR [95%CI]		OR [95%CI]		OR [95%CI]		OR [95%CI]		OR [95%CI]
1 - Current psychotic features	0.93 [0.45–1.91]		1.08 [0.52–2.25]		1.07 [0.48–2.37]		0.95 [0.39–2.33]		0.99 [0.27–3.60]		1.12 [0.31–4.06]		1.10 [0.29–4.14]		1.00 [0.25–4.06]
2 - Significant risk of suicide	1.95 [1.35–2.83]***		1.85 [1.27–2.71]***		1.85 [1.25–2.75]***		2.81 [1.83–4.33]***		1.31 [0.76–2.24]		1.28 [0.73–2.24]		1.32 [0.74–2.36]		**1.93 [1.05–3.56]** *
3 - Alcohol/drug use disorder in the last year	1.16 [0.85–1.59]		1.22 [0.87–1.72]		1.15 [0.77–1.72]		1.28 [0.82–2.00]		**0.54 [0.33–0.86]** *		**0.56 [0.34–0.93]** *		**0.54 [0.32–0.93]** *		0.60 [0.33–1.07]
4 - Comorbid dysthymic disorder	1.26 [0.94–1.70]		1.16 [0.83–1.62]		1.36 [0.95–1.94]		1.34 [0.92–1.93]		1.35 [0.83–2.18]		1.28 [0.76–2.13]		1.46 [0.85–2.49]		1.46 [0.85–2.51]
5 - Score <18 on HAM-D	NA		NA		NA		NA		NA		NA		NA		NA
6 - Past-year comorbid anxiety disorders^b^	1.11 [0.86–1.43]		1.21 [0.93–1.58]		**1.37 [1.02–1.84]** *		1.54 [1.12–2.10]**		1.01 [0.69–1.49]		1.09 [0.74–1.62]		1.22 [0.80–1.86]		1.37 [0.87–2.15]
7 - Episode duration of <4 weeks or >2 years	0.93 [0.72–1.20]		0.86 [0.66–1.13]		0.98 [0.73–1.32]		1.13 [0.82–1.54]		0.70 [0.48–1.00]		**0.65 [0.45–0.96]** *		0.72 [0.48–1.09]		0.82 [0.53–1.25]
8 - Borderline personality disorder	NA		NA		NA		NA		NA		NA		NA		NA
Excluded by at least one criterion	1.08 [0.81–1.42]		1.09 [0.81–1.48]		1.26 [0.87–1.81]		**1.62 [1.06–2.50]** *		**0.62 [0.40–0.96]** *		0.63 [0.40–1.01]		0.71 [0.44–1.16]		0.91 [0.52–1.58]
Eligible participants	0.93 [0.70–1.23]		0.92 [0.68–1.24]		0.80 [0.55–1.14]		**0.62 [0.40–0.95]** *		**1.62 [1.05–2.50]** *		1.59 [0.99–2.49]		1.41 [0.87–2.30]		1.10 [0.63–1.93]

a Derived from Zimmerman et al. [29] (method described in the paper).

b Includes panic disorder, agoraphobia, social anxiety disorder, specific phobia, and generalized anxiety disorder.

Odds ratios were estimated through logistic regression (df = 1).

* p-value <.05; **p-value <.01; ***p-value <.001.

Abbreviations: MDD = Major Depressive Disorder, D(m) = MDD plus Subthreshold Hypomania, D(BP2) = Bipolar 2 depressive disorder, HAM-D = Hamilton Rating Scale for Depression, NESARC = National Epidemiological Survey on Alcohol and Related Conditions, NA = information not available in NESARC.

Models 1, 2, 3 and 4 indicates that subthreshold hypomania was defined as having respectively at least 1, 2, 3, or 4 lifetime concomitant hypomanic probes screening criterion A or B for hypomania.

**Table 4:** Comparisons of Exclusion Rates between Adults with current D(m), Pure MDD, and D(BP2) Seeking Treatment for depression in Typical Clinical Trials of Treatments for DSM-IV MDD by Traditional Eligibility Criteria.

	D(m) vs. Pure MDD								D(m) vs. D(BP2)						
	Model								Model						
	1		2		3		4		1		2		3		4
Traditional efficacy eligibility criteria^a^	OR [95%CI]		OR [95%CI]		OR [95%CI]		OR [95%CI]		OR [95%CI]		OR [95%CI]		OR [95%CI]		OR [95%CI]
1 - Current psychotic features	0.86 [0.37–2.00]		0.99 [0.43–2.29]		0.91 [0.36–2.30]		0.91 [0.34–2.45]		0.92 [0.20–4.28]		1.01 [0.22–4.74]		0.95 [0.19–4.63]		0.95 [0.19–4.82]
2 - Significant risk of suicide	2.37 [1.47–3.81]***		2.49 [1.55–4.02]***		2.20 [1.35–3.57]**		3.41 [1.99–5.86]***		1.57 [0.73–3.39]		1.66 [0.76–3.64]		1.60 [0.72–3.57]		**2.39 [1.03–5.56]** *
3 - Alcohol/drug use disorder in thelast year	1.16 [0.72–1.86]		1.20 [0.72–1.99]		1.10 [0.60–1.99]		0.82 [0.44–1.52]		0.72 [0.35–1.47]		0.74 [0.35–1.56]		0.69 [0.31–1.58]		0.54 [0.24–1.24]
4 - Comorbid dysthymicdisorder	1.07 [0.71–1.62]		1.08 [0.70–1.67]		1.33 [0.82–2.15]		1.31 [0.78–2.20]		1.28 [0.63–2.61]		1.30 [0.63–2.67]		1.53 [0.73–3.24]		1.54 [0.72–3.30]
5 - Score <18 on HAM-D	NA		NA		NA		NA		NA		NA		NA		NA
6 - Past-year comorbid anxietydisorders^b^	1.11 [0.77–1.61]		1.21 [0.82–1.79]		1.26 [0.85–1.89]		1.42 [0.92–2.17]		0.68 [0.37–1.25]		0.73 [0.39–1.35]		0.76 [0.40–1.44]		0.85 [0.45–1.60]
7 - Episode duration of <4 weeksor >2 years	0.82 [0.55–1.23]		0.78 [0.52–1.19]		0.82 [0.52–1.30]		0.82 [0.50–1.32]		0.92 [0.51–1.68]		0.88 [0.48–1.63]		0.91 [0.48–1.74]		0.89 [0.46–1.74]
8 - Borderline personality disorder	NA		NA		NA		NA		NA		NA		NA		NA
Excluded by at least one criterion	1.36 [0.90–2.06]		1.41 [0.92–2.16]		1.48 [0.92–2.40]		1.53 [0.90–2.58]		0.66 [0.31–1.40]		0.68 [0.32–1.46]		0.72 [0.32–1.64]		0.76 [0.32–1.78]
Eligible participants	0.73 [0.48–1.11]		0.71 [0.46–1.08]		0.67 [0.42–1.09]		0.65 [0.39–1.11]		1.52 [0.71–3.24]		1.47 [0.68–3.15]		1.38 [0.61–3.14]		1.32 [0.56–3.10]

a Derived from Zimmerman et al. [29] (method described in the paper).

b Includes panic disorder, agoraphobia, social anxiety disorder, specific phobia, and generalized anxiety disorder.

Odds ratios were estimated through logistic regression (df = 1).

* p-value <.05; **p-value <.01; ***p-value <.001.

Abbreviations: MDD = Major Depressive Disorder, D(m) = MDD plus Subthreshold Hypomania, D(BP2) = Bipolar 2 depressive disorder, HAM-D = Hamilton Rating Scale for Depression, NESARC = National Epidemiological Survey on Alcohol and Related Conditions, NA = information not available in NESARC.

Models 1, 2, 3 and 4 indicates that subthreshold hypomania was defined as having respectively at least 1, 2, 3, or 4 lifetime concomitant hypomanic probes screening criterion A or B for hypomania.

**Figure 1:**
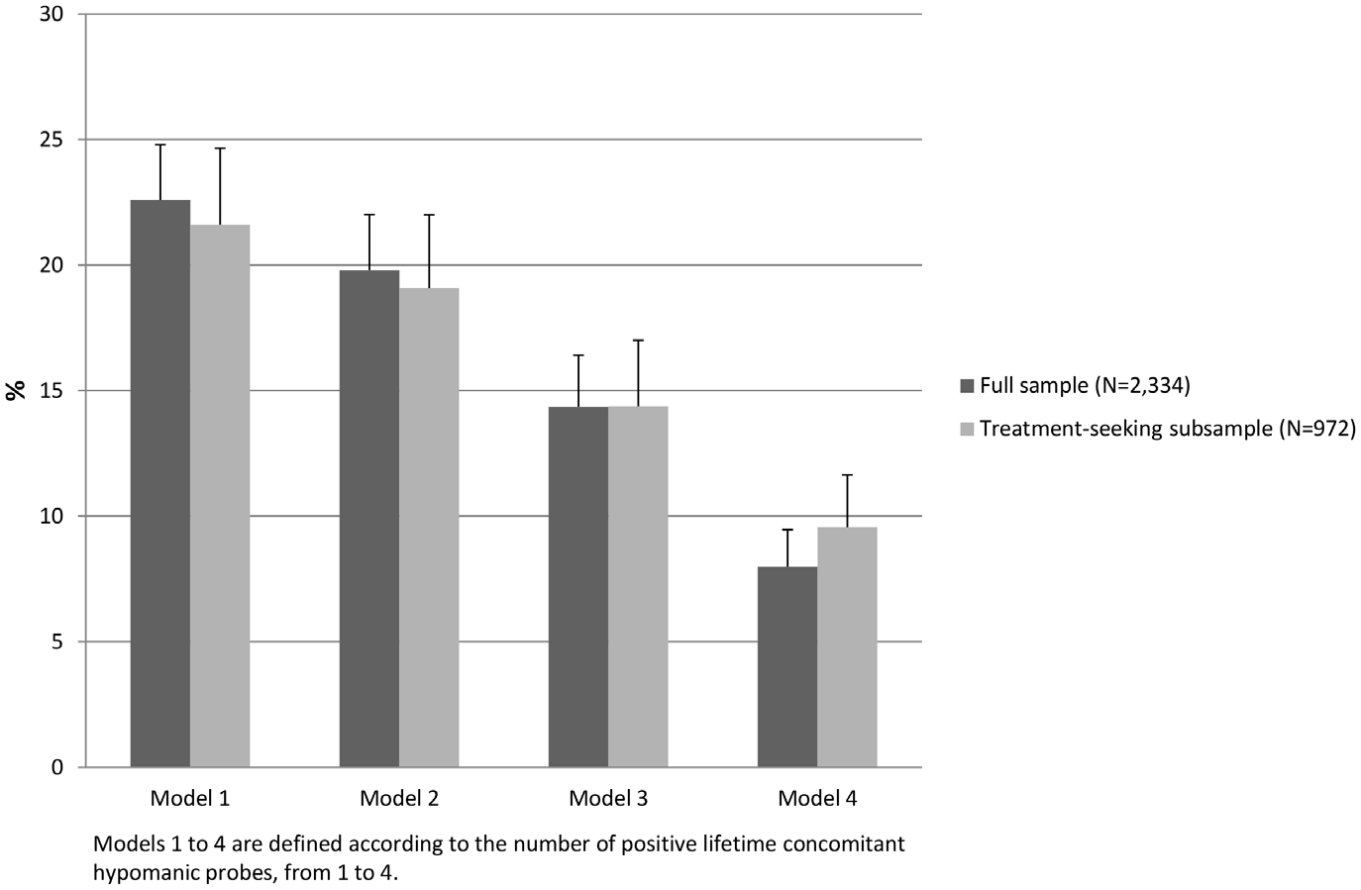
Prevalence rates of individuals with current Major Depressive Disorder plus a lifetime history of Subthreshold Hypomania (D(m)) who would have met classical eligibility criteria in clinical trials for MDD.

In the full sample, a substantial proportion of participants with D(m) would have met inclusion criteria in RCTs with classical eligibility criteria, ranging from 7.98% (SE = 1.48) (when considering a narrow definition of subthreshold hypomania, i.e., at least 4 lifetime concomitant hypomanic probes) to 22.59% (SE = 2.20) with a less stringent threshold (i.e., at least 1 lifetime hypomanic probe). In the subsample of participants who had sought treatment for depression, this percentage would have rose from 9.56% (SE = 2.08) to 21.61% (SE = 3.04) according to the subthreshold bipolar-specifier diagnosis used (Figure 1).

**Figure 2:**
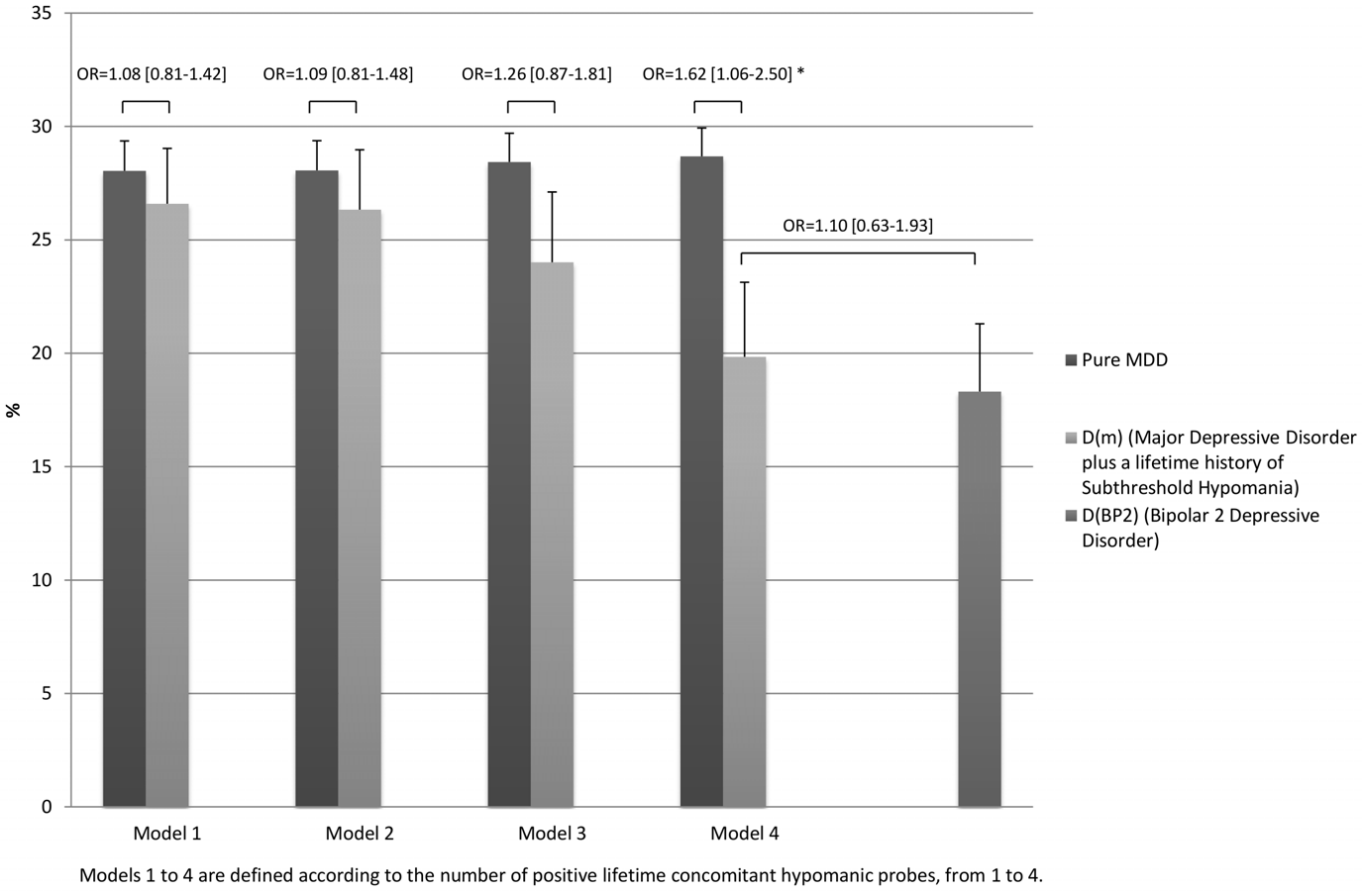
Inclusion rates of individuals with current Pure MDD, D(m), and D(BP2) who would have met typical eligibility criteria of RCTs for MDD.

**Figure 3:**
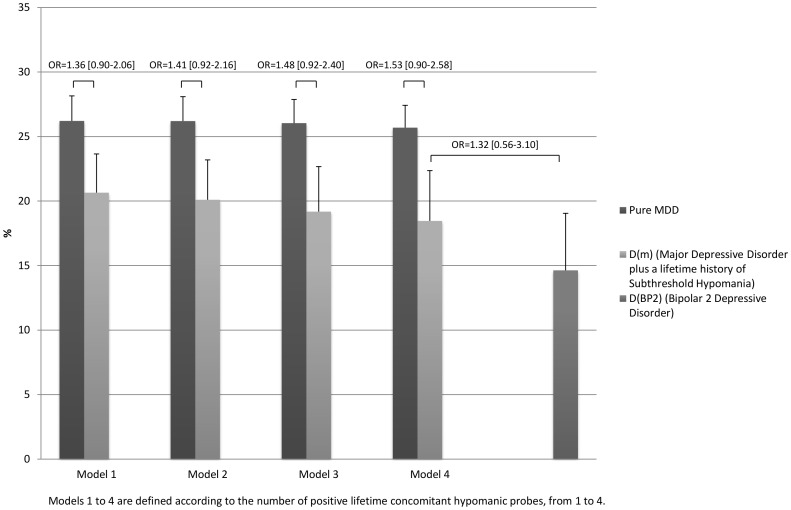
Inclusion rates of individuals with current Pure MDD, D(m), and D(BP2) seeking treatment for depression who would have met typical eligibility criteria of RCTs for MDD.

In the overall sample, the pattern of exclusion rates in individuals with a current diagnosis of MDD plus at least 4 lifetime concomitant hypomanic probes significantly differed from that of participants with pure MDD and was not significantly different from that of individuals with bipolar 2 disorder, except for the criterion “significant risk of suicide” which was significantly higher in participants with D(m) when using the more stringent subthreshold bipolar-specifier (Model 4). Furthermore, overall exclusion rate of participants with MDD plus at least 2 hypomanic probes was not significantly different from that of participants with bipolar 2 disorder (Figure 2). Although a similar pattern of exclusion rates was observed in the treatment-seeking subsample, no significant difference was found in overall exclusion rate between participants with D(m) and those with pure MDD (Figure 3).

## Discussion

To our knowledge, this is the first study attempting to estimate the proportion of adults with MDD plus a lifetime history of subthreshold hypomania (D(m)) that would have been included in clinical trials with traditional eligibility criteria for MDD. We found that the proportion of individuals with D(m) might range from 7.98% to 22.59% in the full sample, and from 9.56% to 21.61% in the treatment-seeking subsample, in typical clinical trials for MDD.

Consistent with prior research [3], [29]–[31], including a recent study examining generalizability of clinical trial results for current major depressive episode using the same database [3], findings indicate that clinical trials tend to exclude, by design, a majority of individuals with current pure MDD. In a typical efficacy trial for MDD, more than 7 out of ten respondents with pure MDD in both the full sample and the treatment-seeking subsample would have been excluded by at least one exclusion criterion. This result supports that clinical trials suffer from impaired external validity since their results may not be readily generalizable to community settings.

Restrictive eligibility criteria used by RCTs at the cost of diminished external validity are justified to reach high internal validity [3]. However, beyond impaired external validity, we found that a substantial proportion of participants that would have been eligible in RCTs for MDD with classical eligibility criteria reported a lifetime history of subthreshold hypomania. In line with prior research supporting the validity of distinguishing depressed individuals with a lifetime history of subthreshold hypomania from those with pure MDD [9], [11], [13], [15], [16], we found that the pattern of exclusion rates (including significant risk of suicide, past-year comorbid anxiety disorders, and overall exclusion rate) in participants with a current diagnosis of MDD plus at least 4 lifetime concomitant hypomanic probes significantly differs from those with pure MDD, whereas it was similar to that in individuals with bipolar 2 disorder, except for the criterion “significant risk of suicide”, which was significantly higher in participants with D(m) when using the more stringent subthreshold bipolar-specifier. Lifetime subthreshold hypomania history among NESARC respondents selectively impacts eligibility on the basis of some exclusion criteria. In the full sample and in the treatment-seeking subsample, a lifetime history of subthreshold hypomania significantly increases at any level of stringency the likelihood of meeting exclusion criterion “significant risk for suicide”, whereas it impacts exclusion for any comorbid anxiety disorder diagnosis only in those endorsing 3 or 4 lifetime concomitant hypomanic probes in the treatment-seeking subsample. In contrast, exclusion rates based on other traditional exclusion criteria appear to be unaffected by D(m) status.

Overall exclusion rate of participants with MDD plus at least 2 hypomanic probes was not significantly different from that of participants with bipolar 2 disorder both in the full sample and in the subsample of participants seeking treatment for depression. With that in mind, including a substantial proportion of individuals with a lifetime history of subthreshold hypomania might be responsible of a selection bias affecting internal validity of trials for MDD. In fact, our results reinforce the possibility that a substantial proportion of individuals with D(m) share similarities with those with bipolar 2 disorder. It was previously suggested that individuals with D(m) have poor response to antidepressants [16], [32]–[36], resembling those with bipolar depression [37]. Such a potential bias selection might therefore lead to an underestimation of antidepressants’ efficacy in placebo-controlled trials and may impact on antidepressants head-to-head trials’ results. As such, despite of the use of restrictive eligibility criteria at the cost of important diminished external validity [3], a substantial proportion of participants with a lifetime history of subthreshold hypomania are nonetheless included, potentially resulting in impaired internal validity.

Furthermore, we found that individuals with D(m) may have greater risk for suicide compared with those with bipolar 2 disorder, when using the more stringent subthreshold bipolar-specifier (Model 4) in the full sample as well as in the treatment-seeking subsample. One possible explanation is that these participants, considered by the current psychiatric classifications as having unipolar depressive disorder, are less likely to benefit from a mood stabilizer compared to those with bipolar 2 disorder, as previously suggested [10].

Some limitations should be considered in interpreting these findings. First, we followed a methodology described by Blanco and colleagues [3] and applied eligibility criteria derived from the work of Zimmerman and colleagues [29] to the NESARC sample. Other conventions might have yield different exclusion estimates. For example, we excluded all individuals with suicide attempt within the last 12 month, considering this question as closest available data to approximate the criterion “significant risk of suicide”. In addition, the 12-month timeframe used by the AUDADIS-IV when assessing the presence of “current” symptoms could have led to an overestimation of the exclusion rate and the proportion of individuals potentially eligible in RCTs for MDD. However, the percentage of excluded participants was high and consistent with those observed in earlier research [3], [29]–[31], suggesting that commonly applied criteria are likely to exclude a majority of individuals with pure MDD. Nevertheless, development of procedures to operationalize eligibility criteria selection might help refine future generalizability estimates.

Second, in absence of consensus subthreshold bipolar-specifier diagnosis [11], [19], we defined four models including different subthreshold bipolar-specifier diagnoses. We thus identified participants with MDD plus a lifetime history of subthreshold hypomania based on the lifetime presence during over one week of at least one, two, three or four concomitant hypomanic probes, screening criterion A or B for hypomania. These definitions were somewhat arbitrary and other conventions might have lead to different results. Furthermore, these narrow definitions, both in terms of the choice of hypomanic symptoms and their duration, could have led to underestimate the proportion of depressed participants with a lifetime subthreshold hypomania [10]. At last, it has to be raised that the way models where compared in the present work implicitly accept the notion that with more subthreshold positive probes of hypomanic symptoms, the risk to reflect bipolar disorder is increasing. We would like to suggest that a consensus subthreshold bipolar-specifier diagnosis would be helpful to operationalize eligibility assessment of subthreshold hypomania in clinical trials for major depressive disorder [10], [11].

Third, two exclusion criteria were not available in the NESARC and may theoretically have led to underestimate the proportion of participants excluded in clinical trials. For example, Zimmerman et al. [38] have estimated that a score lower than 14 on HAM-D would exclude 32% to 47% of individuals with MDD. However, the percentage of excluded participants was high and consistent with those observed in earlier research [3], [29]–[31], supporting that these two missing criteria may have little impact on the overall exclusion rate.

Fourth, as previously indicated by Blanco and colleagues [3], our approach focuses on the *a priori* eligibility of participants and was based on national epidemiological data. It provides no information on individuals who actually enter those studies. In this way, we estimate an upper bound of the generalizability of clinical trials. Particularly, a substantial proportion of potential eligible individuals may be unwilling to participate [39]. Furthermore, the likelihood of entering a trial may be influenced by several factors, including anxiety, extroversion, work satisfaction, and performance measures [40].

Fifth, although similar pattern of exclusion rates was observed in the treatment-seeking subsample compared to that of the full sample, no significant difference was found in overall exclusion rate between participants with D(m) and those with pure MDD. Although this result might be due to a lack of statistical power and a floor effect, it is possible that D(m) status may exert less impact on eligibility in individuals seeking treatment for depression.

At last, severity and clinical significance of each disorder are determined by the AUDADIS-IV at the syndromal rather than symptom level. In addition, AUDADIS-IV reliability for diagnoses of anxiety disorders is only fair [25].

Despite these limitations, this study suggests that the current design of clinical trials for MDD suffers from impaired external validity as well as potential impaired internal validity due to the inclusion of a substantial proportion of individuals with D(m), that may differ from those with pure MDD but not from those with bipolar 2 disorder. We want to emphasize the need of assessing lifetime hypomanic symptoms in eligibility assessment for RCTs for MDD. Individuals with at least 4 lifetime concomitant hypomanic probes might be more accurately excluded from RCTs for MDD and considered as having bipolar 2 disorder, and those with at least 2 hypomanic probes should be systematically subject to a sensitivity analysis to test the robustness of trials’ results. Future studies would benefit from evaluating the influence of individuals with a lifetime history of subthreshold hypomania on placebo-controlled and antidepressants head-to-head clinical trials’ results, as well as efficacy and adverse effects of antidepressants in these patients.

## References

[1] Bucher HC, Guyatt GH, Cook DJ, Holbrook A, McAlister FA (1999) Users’ guides to the medical literature: XIX. Applying clinical trial results. A. How to use an article measuring the effect of an intervention on surrogate end points. Evidence-Based Medicine Working Group. Jama 282: 771–778. 10463714 10.1001/jama.282.8.771

[2] Oxman AD, Sackett DL, Guyatt GH (1993) Users’ guides to the medical literature. I. How to get started. The Evidence-Based Medicine Working Group. Jama 270: 2093–2095. 8411577

[3] Blanco C, Olfson M, Goodwin RD, Ogburn E, Liebowitz MR, et al (2008) Generalizability of clinical trial results for major depression to community samples: results from the National Epidemiologic Survey on Alcohol and Related Conditions. J Clin Psychiatry 69: 1276–1280. 18557666 10.4088/jcp.v69n0810

[4] Hoertel N, Le Strat Y, De Maricourt P, Limosin F, Dubertret C (2012) Are subjects in treatment trials of panic disorder representative of patients in routine clinical practice? Results from a national sample. J Affect Disord. doi: 10.1016/j.jad.2012.09.023. 10.1016/j.jad.2012.09.023 23084184

[5] Hoertel N, Le Strat Y, Lavaud P, Dubertret C, Limosin F (2013) Generalizability of clinical trial results for bipolar disorder to community samples. J Clin Psychiatry. doi:10.4088/JCP.12m07935. 10.4088/JCP.12m07935 23561233

[6] Dzewaltowski DA, Estabrooks PA, Klesges LM, Bull S, Glasgow RE (2004) Behavior change intervention research in community settings: how generalizable are the results? Health Promot Int 19: 235–245. 15128715 10.1093/heapro/dah211

[7] Ustun TB, Ayuso-Mateos JL, Chatterji S, Mathers C, Murray CJ (2004) Global burden of depressive disorders in the year 2000. Br J Psychiatry 184: 386–392. 15123501 10.1192/bjp.184.5.386

[8] Wittchen HU, Jacobi F (2005) Size and burden of mental disorders in Europe–a critical review and appraisal of 27 studies. Eur Neuropsychopharmacol 15: 357–376. 15961293 10.1016/j.euroneuro.2005.04.012

[9] Angst J, Cui L, Swendsen J, Rothen S, Cravchik A, et al (2010) Major depressive disorder with subthreshold bipolarity in the National Comorbidity Survey Replication. Am J Psychiatry 167: 1194–1201. 20713498 10.1176/appi.ajp.2010.09071011 PMC3145248

[10] Hoertel N, Le Strat Y, Angst J, Dubertret C (2012) Subthreshold Bipolar Disorder in a U.S. National Representative Sample: Prevalence, Correlates and Perspectives for Psychiatric Nosography. J Affect Disord. doi: 10.1016/j.jad.2012.09.016. 10.1016/j.jad.2012.09.016 23040874

[11] Angst J, Gamma A, Benazzi F, Ajdacic V, Eich D, et al (2003) Toward a re-definition of subthreshold bipolarity: epidemiology and proposed criteria for bipolar-II, minor bipolar disorders and hypomania. J Affect Disord 73: 133–146. 12507746 10.1016/s0165-0327(02)00322-1

[12] Lewinsohn PM, Klein DN, Seeley JR (1995) Bipolar disorders in a community sample of older adolescents: prevalence, phenomenology, comorbidity, and course. J Am Acad Child Adolesc Psychiatry 34: 454–463. 7751259

[13] Merikangas KR, Herrell R, Swendsen J, Rossler W, Ajdacic-Gross V, et al (2008) Specificity of bipolar spectrum conditions in the comorbidity of mood and substance use disorders: results from the Zurich cohort study. Arch Gen Psychiatry 65: 47–52. 18180428 10.1001/archgenpsychiatry.2007.18

[14] Szadoczky E, Papp Z, Vitrai J, Rihmer Z, Furedi J (1998) The prevalence of major depressive and bipolar disorders in Hungary. Results from a national epidemiologic survey. J Affect Disord 50: 153–162. 9858075 10.1016/s0165-0327(98)00056-1

[15] Zimmermann P, Bruckl T, Nocon A, Pfister H, Lieb R, et al (2009) Heterogeneity of DSM-IV major depressive disorder as a consequence of subthreshold bipolarity. Arch Gen Psychiatry 66: 1341–1352. 19996039 10.1001/archgenpsychiatry.2009.158

[16] Angst J, Azorin JM, Bowden CL, Perugi G, Vieta E, et al (2011) Prevalence and characteristics of undiagnosed bipolar disorders in patients with a major depressive episode: the BRIDGE study. Arch Gen Psychiatry 68: 791–798. 21810644 10.1001/archgenpsychiatry.2011.87

[17] Judd LL, Akiskal HS (2003) The prevalence and disability of bipolar spectrum disorders in the US population: re-analysis of the ECA database taking into account subthreshold cases. J Affect Disord 73: 123–131. 12507745 10.1016/s0165-0327(02)00332-4

[18] Fiedorowicz JG, Endicott J, Leon AC, Solomon DA, Keller MB, et al (2010) Subthreshold hypomanic symptoms in progression from unipolar major depression to bipolar disorder. Am J Psychiatry 168: 40–48. 21078709 10.1176/appi.ajp.2010.10030328 PMC3042431

[19] Angst J, Gamma A, Bowden CL, Azorin JM, Perugi G, et al.. (2011) Diagnostic criteria for bipolarity based on an international sample of 5,635 patients with DSM-IV major depressive episodes. Eur Arch Psychiatry Clin Neurosci. 10.1007/s00406-011-0228-0 21818629

[20] Blanco C, Olfson M, Okuda M, Nunes EV, Liu SM, et al (2008) Generalizability of clinical trials for alcohol dependence to community samples. Drug Alcohol Depend 98: 123–128. 18579319 10.1016/j.drugalcdep.2008.05.002 PMC3755733

[21] Hoertel N, Le Strat Y, Blanco C, Lavaud P, Dubertret C (2012) Generalizability of Clinical Trial Results for Generalized Anxiety Disorder to Community Samples. Depress Anxiety 29: 614–620. 22495990 10.1002/da.21937

[22] Grant BF, Harford TC, Dawson DA, Chou PS, Pickering RP (1995) The Alcohol Use Disorder and Associated Disabilities Interview schedule (AUDADIS): reliability of alcohol and drug modules in a general population sample. Drug Alcohol Depend 39: 37–44. 7587973 10.1016/0376-8716(95)01134-k

[23] Grant BF, Stinson FS, Dawson DA, Chou SP, Dufour MC, et al (2004) Prevalence and co-occurrence of substance use disorders and independent mood and anxiety disorders: results from the National Epidemiologic Survey on Alcohol and Related Conditions. Arch Gen Psychiatry 61: 807–816. 15289279 10.1001/archpsyc.61.8.807

[24] Hasin DS, Goodwin RD, Stinson FS, Grant BF (2005) Epidemiology of major depressive disorder: results from the National Epidemiologic Survey on Alcoholism and Related Conditions. Arch Gen Psychiatry 62: 1097–1106. 16203955 10.1001/archpsyc.62.10.1097

[25] Grant BF, Dawson DA, Stinson FS, Chou PS, Kay W, et al (2003) The Alcohol Use Disorder and Associated Disabilities Interview Schedule-IV (AUDADIS-IV): reliability of alcohol consumption, tobacco use, family history of depression and psychiatric diagnostic modules in a general population sample. Drug Alcohol Depend 71: 7–16. 12821201 10.1016/s0376-8716(03)00070-x

[26] Canino G, Bravo M, Ramirez R, Febo VE, Rubio-Stipec M, et al (1999) The Spanish Alcohol Use Disorder and Associated Disabilities Interview Schedule (AUDADIS): reliability and concordance with clinical diagnoses in a Hispanic population. J Stud Alcohol 60: 790–799. 10606491 10.15288/jsa.1999.60.790

[27] Chatterji S, Saunders JB, Vrasti R, Grant BF, Hasin D, et al (1997) Reliability of the alcohol and drug modules of the Alcohol Use Disorder and Associated Disabilities Interview Schedule–Alcohol/Drug-Revised (AUDADIS-ADR): an international comparison. Drug Alcohol Depend 47: 171–185. 9306043 10.1016/s0376-8716(97)00088-4

[28] Hasin D, Carpenter KM, McCloud S, Smith M, Grant BF (1997) The alcohol use disorder and associated disabilities interview schedule (AUDADIS): reliability of alcohol and drug modules in a clinical sample. Drug Alcohol Depend 44: 133–141. 9088785 10.1016/s0376-8716(97)01332-x

[29] Zimmerman M, Mattia JI, Posternak MA (2002) Are subjects in pharmacological treatment trials of depression representative of patients in routine clinical practice? Am J Psychiatry 159: 469–473. 11870014 10.1176/appi.ajp.159.3.469

[30] Gaynes BN, Warden D, Trivedi MH, Wisniewski SR, Fava M, et al (2009) What did STAR*D teach us? Results from a large-scale, practical, clinical trial for patients with depression. Psychiatr Serv 60: 1439–1445. 19880458 10.1176/ps.2009.60.11.1439

[31] Zimmerman M, Chelminski I, Posternak MA (2005) Generalizability of antidepressant efficacy trials: differences between depressed psychiatric outpatients who would or would not qualify for an efficacy trial. Am J Psychiatry 162: 1370–1372. 15994721 10.1176/appi.ajp.162.7.1370

[32] Keck PE Jr, Kessler RC, Ross R (2008) Clinical and economic effects of unrecognized or inadequately treated bipolar disorder. J Psychiatr Pract 14 Suppl 231–38. 18677197 10.1097/01.pra.0000320124.91799.2a

[33] Licht RW, Vestergaard P, Brodersen A (2008) Long-term outcome of patients with bipolar disorder commenced on lithium prophylaxis during hospitalization: a complete 15-year register-based follow-up. Bipolar Disord 10: 79–86. 18199244 10.1111/j.1399-5618.2008.00499.x

[34] Cruz N, Sanchez-Moreno J, Torres F, Goikolea JM, Valenti M, et al (2010) Efficacy of modern antipsychotics in placebo-controlled trials in bipolar depression: a meta-analysis. Int J Neuropsychopharmacol 13: 5–14. 19638254 10.1017/S1461145709990344

[35] Angst J (2007) The bipolar spectrum. Br J Psychiatry 190: 189–191. 17329735 10.1192/bjp.bp.106.030957

[36] Seemuller F, Severus E, Moller HJ, Riedel M (2009) Antidepressants and suicidality in younger adults–is bipolar illness the missing link? Acta Psychiatr Scand 119: 166; author reply 167. 10.1111/j.1600-0447.2008.01328.x 19120044

[37] Ghaemi SN (2008) Why antidepressants are not antidepressants: STEP-BD, STAR*D, and the return of neurotic depression. Bipolar Disord 10: 957–968. 19594510 10.1111/j.1399-5618.2008.00639.x

[38] Zimmerman M, Chelminski I, Posternak MA (2004) Exclusion criteria used in antidepressant efficacy trials: consistency across studies and representativeness of samples included. J Nerv Ment Dis 192: 87–94. 14770052 10.1097/01.nmd.0000110279.23893.82

[39] Melberg HO, Humphreys K (2010) Ineligibility and refusal to participate in randomised trials of treatments for drug dependence. Drug Alcohol Rev 29: 193–201. 20447229 10.1111/j.1465-3362.2009.00096.x

[40] Mavissakalian MR, Guo S (2002) Predictors of entering a long-term drug treatment study of panic disorder. Compr Psychiatry 43: 88–94. 11893985 10.1053/comp.2002.30803

